# The Magic Cabinet

**Published:** 1881-09

**Authors:** 


### THE MAGIC CABINET.

Many ingenious illusions have been contrived, depending on the laws of reflection from plane surfaces. Among these is the well-known one devised for theaters by the physicist Robin, and which at one time attracted great attention under the name of "Pepper's Ghost." This spectral illusion is produced by reflections from a large sheet of unsilvered plate glass, which is so arranged that the actors on the stage are seen through it, while other actors placed in strong illumination, and out of direct view of the spectators, are seen by reflections in it, and appear as ghosts on the stage. But one of the most striking applications of mirrors for the amusement of an audience is undoubtedly that seen in the contrivance represented in the annexed figure and known as the *magic cabinet.* Some years ago an exhibition of this kind drew large audiences of curiosity seekers to witness it, both in Paris and in a large number of other cities. The visitor, on gazing into a small cabinet, which no one was allowed to enter, saw a small three-legged table, on which lay a large plate containing a human head. This head, which was apparently that of a decapitated person, moved its eyes, made grimaces, and talked. Although the spectators believed that they saw an empty space beneath the table, the individual to whom the head belonged was really seated there, his body being hidden by two vertical glass mirrors fitted between the legs of the table at an angle of forty-five degrees with the two side walls. The whole was so arranged that these two walls coincided with the visible portions of the wall in the rear of the cabinet. The three walls were painted of a homogeneous color, and the illusion being enhanced by the feeble light employed, the effect was most remarkable. Had some spectator, however, thrown a stone between the legs of the table, a crash of glass would have followed and at once have unveiled the mystery.—*La Nature.*

